# ‘I probably wouldn’t want to talk about anything too personal’: A qualitative exploration of how issues of privacy, confidentiality and surveillance in the home impact on access and engagement with online services and spaces for care-experienced young people

**DOI:** 10.1177/03085759231203019

**Published:** 2023-10-19

**Authors:** Lorna Stabler, Emily Cunningham, Dawn Mannay, Maria Boffey, Aimee Cummings, Brittany Davies, Charlotte Wooders, Rachael Vaughan, Rhiannon Evans

**Affiliations:** Cardiff University, UK; University of Glasgow, UK; Cardiff University, UK; Cardiff University, UK; Cardiff University, UK; Cardiff University, UK; The Fostering Network in Wales, UK; Cardiff University, UK; Cardiff University, UK

**Keywords:** Mental health, Covid-19, care-experienced young people, foster care, children in care, mental health services

## Abstract

This paper draws on a qualitative interview-based study that explored online mental health and wellbeing interventions and services for care-experienced young people. The study involved young people (*n* = 4), foster carers (*n* = 8), kinship carers (*n* = 2) and social care professionals (*n* = 9) in Wales, UK. The paper reflects on the complexities of online communication in the space of ‘the home’. It documents the ways in which care-experienced young people’s living arrangements can restrict access to services and complicate confidentiality within portals to the virtual world, creating an environment where young people and their carers ‘wouldn’t want to talk about anything too personal’. Drawing on data generated in a study focused on services and interventions to support the mental health and wellbeing of care-experienced children and young people, the paper considers privacy, confidentiality and surveillance in the home and reflects on how associated relational practices impact on care-experienced young people. While the data discussed in this paper was generated during the Covid-19 pandemic, its findings have implications for how care-experienced young people and their carers can be supported to engage with the digital world in the future.

## Plain Language Summary

When mental health services are delivered online, they are usually accessed from home. Home can be different for young people who grow up in care, with records kept about their day-to-day activities, a lack of access to the digital world and policy governing how they are able to live their lives. We spoke with young people, foster carers and practitioners about what it is like for young people to access mental health services online. The paper discusses how the private home lives of care-experienced children and young people impacts on how they are able to access much needed mental health services. We look at the need to protect the freedom of children in care and care-experienced young people to take risks and access digital spaces like other children and teenagers do, while also recognising the reality of the pressures on practitioners and carers to protect looked after children from harm.

## Background

In March 2020, the Covid-19 pandemic instigated a nationwide lockdown in Wales (Institute for Government, 2020), and its impacts were felt across the UK and internationally. Mental health in the UK general population deteriorated compared with pre-pandemic trends (Pierce et al., 2020) and the pandemic’s social distancing restrictions led to increased cases of anxiety, depressive symptoms and behavioural difficulties (Williams, 2020). While the Covid-19 pandemic has had similar effects on all young people, its restrictions had differential outcomes for already marginalised communities (Patel et al., 2020; Withers, 2020).

The impact of the pandemic on the mental health and wellbeing of care-experienced young people has been reported across the UK. In Northern Ireland, Kelly and colleagues interviewed 24 care leavers who discussed how it ‘exacerbated already complex issues, sometimes resulting in elevated symptoms and psychological distress’ (2020: 21). In Wales, Roberts and colleagues’ (2021a; 2021b) study with 21 care leavers illustrated how extended periods of confinement in their homes and a lack of routine resulted in their mental health difficulties becoming more pronounced. In the Irish context, Gilligan and colleagues (2022) spoke with 16 care leavers aged 18–27 who discussed how the pandemic brought ‘big’ and ‘small’ disruptions, which impacted on their mental health.

This context necessitated an increase in the delivery of mental health services, which involved a large-scale move of services to remote delivery, online or via the telephone. The use of such methods within the delivery of services to children and young people in and leaving care was limited before the pandemic and subject to concerns about efficacy, accessibility, safety and feasibility, often used reactively rather than proactively (Pascoe, 2023). However, where access to online contact has been facilitated, practitioners have emphasised that young people have benefitted from social media and digital communication (Munro et al., 2021; Roberts et al., 2020).

Nonetheless, these changes, from in-person to remote delivery methods, intersected with a range of challenging situations faced by care-experienced children and young people during and outside of the pandemic. Care-experienced children, young people and care leavers reported isolation, digital exclusion, financial instability and precarious access to support and services in the UK (Leicestershire Cares, 2021; NYAS, 2020; O’Higgins, Canning and Taylor, 2020) and internationally (Rosenberg et al., 2022; Shpiegel et al., 2022). Additionally, the digital divide has uneven impacts, and can limit access to digital spaces for care-experienced young people (McGhee and Roesch-Marsh, 2020).

Barriers to digital access have been well documented in the existing literature. Yet, the physical space of the home, which acts as care-experienced young people’s portal to digital spaces, has drawn little academic attention. Studies have focused on the challenges of being at home and disruptions to routine during the pandemic (Roberts et al., 2021a; Whitt-Woosley, Sprang and Eslinger, 2022), but there has been little consideration of privacy, confidentiality and surveillance in relation to the interaction of online/home spaces in the context of service delivery. This acknowledgment is important as the concept of ‘home’ is often complicated for care-experienced children and young people.

On entering the care system, care-experienced young people are the focus of national policies and interventions. Their lives are often paradoxically characterised by high levels of state scrutiny (Shepherd et al., 2020) but chronically poor and inconsistent state social and mental health support (Evans et al., 2021). Alongside this, carers in the home are expected to play a dual role of emotional and familial caregiver as well as paid professional held to account by authorities (Schofield et al., 2013; Hollett, Hassett and Lumsden, 2022). Additionally, children and young people rarely occupy positions of power within the domestic arena, and this is particularly the case for those in care whose position within the ‘family’ and the ‘home’ can be precarious (Mannay et al., 2017; Ward, 2009).

Access to digital spaces for care-experienced young people has long been debated, with access to social media in particular constrained by moral panics around safeguarding (Hammond, Cooper and Jordan, 2018) and complexities regarding court- and social work-mandated restrictions of children and young people’s contact with their birth family while in care (Simpson, 2016). However, the Covid-19 pandemic transformed the domestic space with education, health, social and mental health provision entering the home through digital communication. This presented new challenges around privacy, confidentiality, surveillance and negotiation within foster- and kinship-caring relationships (Roberts et al., 2021a), and young people’s access to digital spaces for social and support purposes was mediated through rules and restrictions imposed from both within and outside the home.

The online world is not a hermeneutically separate space, and young people need to find portals to the virtual world often within the ‘privacy’ of the bedroom. The bedroom does not exist in isolation: it is a space shaped by the household in which the bedroom exists (Lincoln, 2012). Home is often seen as a type of sanctuary: a private social space which is particularly impervious to outside observation (Lincoln, 2012). Yet, for care-experienced young people, the home is also a space within the wider arenas of public policy, social work and discourses of difference, stigma and visibility. When the home is occupied by care-experienced young people its walls become more porous, with visits (digital or otherwise) from social workers and other health and social care professionals. These visits can extend from the more public areas of kitchens and living rooms to bedroom spaces, which would otherwise afford an element of privacy from uninvited outsiders. Foster carers are also required to record the everyday lives of those in their care, which are shared with social workers. This can make privacy a rare commodity, which needs to be continually negotiated, and the space where one can be *in privacy* is not only material (a room in the home) but also digital. Accordingly, it is important to challenge the notion of an online–offline divide (Ringrose, 2013) and to examine how ‘real’ life experiences mediate online engagements.

## Methods

A wider study (detailed in Mannay et al., 2022) sought to understand the provision of mental health and wellbeing services and interventions for care-experienced children and young people from the perspectives of young people, foster and kinship carers and practitioners, considering in-person (face-to-face), remote (online and telephone) and blended (a combination of the former) delivery (Mannay et al., 2022). The study addressed the following research questions:

1. How did the Covid-19 pandemic impact on the provision of mental health and wellbeing services and interventions for care-experienced young people?
2. What are the strengths and challenges of online, telephone, face-to-face and blended services and interventions in supporting mental health and wellbeing?
3. What improvements can be made in the format, functioning, delivery, acceptability and accessibility of mental health and wellbeing services and interventions?

In line with valuing the perspectives of ‘experts by experience’ (Staples et al., 2019), a care-experienced young person worked on the research team and consultations were held with care-experienced young people to inform the study design. There was a formal collaboration with The Fostering Network in Wales to shape the project with the input of foster and kinship carers.

Young people were recruited via The Fostering Network in Wales, Voices from Care Cymru and The Roots Foundation Wales, to ensure both the appropriateness of participants to take part in the project and continued access to support for young people and carers. Carers and professionals were recruited via The Fostering Network in Wales.

The study included three care-experienced young people and one young person who was the biological child of a foster carer. Young people were aged between 18 and 27. Nine health and social care professionals, eight foster carers and two kinship carers also contributed to the project. All participants resided in Wales.

A qualitative approach was taken, with one-to-one or small group interviews held between April and July 2021 during the restrictions of the Covid-19 pandemic. Topic guides covered experiences of using remote, blended and in-person services and recommendations for developing and delivering services in the future. Carer and professional interview guides included an additional focus on risk assessments and safeguarding procedures.

As with other studies involving care-experienced young people in the pandemic (Roberts et al., 2021a; 2021b; Sprecher et al., 2021), interviews were conducted remotely via online conferencing platforms (Zoom and Microsoft Teams). Interviews with young people were undertaken by one care-experienced researcher and another researcher from the team. Interviews with carers and professionals were conducted by a single member of the research team. Young people’s interviews ranged from 29 to 64 minutes, with a total of three hours, 21 minutes. Carer interviews ranged from 18 to 68 minutes, a total of seven hours, 31 minutes. Interviews with professionals ranged from 37 to 58 minutes, a total of five hours, 33 minutes, and included one interview with a small group (*n* = 3). Interviews were transcribed by a professional transcribing service generating 172,111 words of transcript, which were thematically analysed in relation to the project research questions (Mannay et al., 2022).

Following data generation and analysis, the study team met with three advisory groups to discuss the findings of the study and inform the recommendations for policy and practice. These groups included foster and kinship carers (*n* = 10) and care-experienced young people (*n* = 8).

A secondary analysis then explored themes identified in the initial analysis around privacy and confidentiality in more depth. A coding tree based on axial coding (Allen, 2017; Strauss and Corbin, 1998) was developed in NVivo software, focusing on identifying themes and relationships between themes across the data. Researchers coded a cross-section of the interview schedules, adding new themes inductively and then comparing coding. Themes were consolidated through discussion in meetings. Narratives for each overarching theme were written, using the structure of the coding frame.

The project team had combined experiences of working with care-experienced young people, carers and health and social care professionals. Accordingly, they were aware of the associated sensitivities and best practice ethical protocols. Ethical approval for the study was provided by the Social Research Ethics Committee at Cardiff University.

## Findings

The findings are presented in relation to three key themes. The first, ‘Being and feeling at home’, relates to the context of the fostering home and some of the particularities within this space that blur the lines between private home space and public scrutinised environment. The second, ‘Privacy, confidentiality and digital communication within the home’, highlights how the nature of the physical space of the fostering home mediates the ways in which children and young people are able to access digital spaces. The third, ‘Navigating the digital world within the home’, explores young people’s responses to this interplay between physical and digital spaces.

### Being and feeling at home

Care-experienced young people’s ‘homes’ can be complex and challenging places to live. Participants’ accounts highlighted how intricate relationships impacted their sense of feeling ‘at home’. For some, the home was conceptualised as ‘unsafe’, with some carers feeling anxious at home because young people made the space ‘challenging’:

… My anxiety has been pretty bad since he’s been back, because I’m scared he’s going to be bringing stuff [illegal substances] into the house, especially with the babies here, and I, I get, my anxieties are bad, thinking that he may be fibbing about, that he’s not being honest with us. (Carer)

This points to the idea that relationality, interdependence, connection (Williams, 2004) and the ‘bi-directionality’ (Lipscombe, Moyers and Farmer, 2004) of relationships within these homes may be different to those in other parent–child relationships. While children and young people in care have a ‘home’, whether they are allowed to remain in the home may be more contingent on their behaviour and other factors related to fostering than for other children in the population (Leathers et al., 2019). This could have an impact on the relationship that a young person has with their home and whether they feel it is a place of safety and security.

For some, the home was associated with difficult memories, and one young person discussed how returning home from independent living during the Covid-19 pandemic negatively impacted their emotional wellbeing:

When I went back [home], then it sort of like brought up a lot of things, and like being stuck there again, like in the beginning, you know, like that was like, like not triggering, but like, it was just like, not a very nice like nostalgia. (Young person)

During the pandemic, the purpose of the home expanded to include activities, such as therapy and meetings with social workers, that were previously in-person and often outside of the home. These activities infiltrated the home, predominately via remote communication. This presented logistical and emotional challenges, and carers documented how specific domestic spaces acquired a negative connotation for young people when they became the site of difficult emotional conversations:

So, I had one young person who was working with a psychologist. And it was very, very difficult, because it was all happening in our space. And even though I move around the different rooms, trying [different rooms] … ‘Right, okay, let’s …’ So, you had to deal with those memories or whatever in that room. ‘Let’s not use that room.’ [example of carer speaking to young person] (Carer)

Accordingly, dealing with traumatic memories in the home disrupted feelings of comfort, safety and domesticity that may have previously defined this space for young people. Being confined in the home during lockdown also impacted on the ability of carers to find private time and space away from their caring role and to get the support that they and their family needed:

But the support really, there was no support, because nobody was allowed to come into your home, nobody was allowed to take the children out. So that was it, you’d get … you’d get a phone call, and that was the level of support really. (Carer)

While ordinarily family members have interactions outside the home, relocating these online during the pandemic meant that everyone was in each other’s space all of the time, increasing tensions in the home environment (see also Kelly et al., 2020) and limiting privacy and confidentiality (Cummings, 2022).

### Risk versus privacy within the home

Within kinship, foster and residential care, there is a complicated blurring of public and private, professional and personal boundaries inherent in the way the care relationship is constructed and scrutinised by external authorities. This has been researched in the context of adolescence (see Lincoln, 2012) but the added complexity of the ‘care’ environment was evident in the accounts of carers and young people. This can serve as a reminder to both a carer and young person that their home is not a ‘normal’ private family home. One foster carer talked about needing to go through laborious sign-off processes with social workers when the young person they cared for wanted to have a sleepover:

You know, it was just, she wanted to be a normal teenager. And that’s the issue with social services sometimes, they don’t see the bigger picture … they walk in with an attitude, that she is a horrible child, and that’s not what we see on a day-to-day basis. (Carer)

Sinclair, Wilson and Gibbs (2001) advocated for the right to an ‘ordinary life’ for children in care, and the *Safer Caring* report (Slade, Bhullar ands Boffey, 2012) recommended a ‘risk sensible’ rather than a ‘risk averse’ approach. However, organising sleepovers with friends remains problematic (Narey and Owers, 2018) despite guidance that authority is delegated to foster carers wherever possible to make day-to-day decisions:

Looked after children should as far as possible be granted the same permissions to take part in normal age-appropriate peer activities (such as sleepovers) as would reasonably be granted by the parents of their peers. (Welsh Government, 2018: 47)

The findings of this study highlight that there are still challenges in practice in managing who makes these decisions for children in care and what can remain part of private family life.

Moreover, the levels of surveillance and scrutiny that carers experience create expectations to respond to situations differently to other family households. One foster carer highlighted that a mark or bruise as a result of playing outside requires a foster carer to declare this to social services and to create a record of the event. This awareness of scrutiny could lead to risk aversion among carers in terms of the freedoms that young people are allowed:

It’s really frustrating for carers that we have to cover ourselves so much. You know, the slightest mark, I have to make sure I’ve written it down, I have to ring the social worker, make sure they know … to try and cover yourself … They’re going to get bruises, but they’re kids … and the scooters, and they end up on their bum and you laugh and you think, but that’s normal family life. (Carer)

Monitoring and reporting of risk can be more related to process than concern for the safety of the child. This has been referred to as ‘risk-to-self averse’ in child protection, in recognition that much of this process is about avoiding blame rather than preventing harm (Munro, 2019). This presents challenges for everyone in the home in terms of maintaining some semblance of ‘normal’ family life and privacy. This surveillance from external authorities seeps into the home, which creates tensions between carers and young people around the right to privacy.

Monitoring and surveillance also characterise the digital world. Carers and young people discussed the importance of negotiating and compromising on online access and recognised that there are times that a young person may need to be in a different space to a carer:

If you want to do something, talk to people you know, and, you know, when they’re on the internet, they don’t want me in the room anyway, so ‘You’re talking, who’s this?’ [checking with young person who they are talking to]. ‘Yeah, fine’. So it’s open communication. (Carer)

This needed to be negotiated based on what was appropriate for the specific young person, in relation to their age, circumstances and any additional learning needs. The need for this negotiation and trust was highlighted by one of the young people:

I recognise that concern, but… if someone has got mental health problems, why you wouldn’t want them to have access to like, the internet, in case they find something and then [are] encouraged or whatever [e.g. to self-harm]? But, I mean, it’s… I think you’ve just got to put that faith in them, and with someone younger, I think they’d probably just access online things, like apps… and websites, where they can like chat, and go in chat rooms and things. (Young person)

In some cases, the difference in relationship that young people might have with digital spaces, and their desire for privacy, did not seem to be fully recognised by carers, when the rules around using digital devices were household wide:

The phones are not allowed in the bedroom, I don’t take mine up, so why should they? You know, that’s the house rule… And it’s trying to keep people as safe as is humanly possible. (Carer)

This regulation of online access was mirrored in the accounts of young people reflecting on the rules that were in place while they were in care:

I wasn’t allowed any social media, so it felt kind of weird… everyone was on social media, and when I finally, you know, could have like group chats with my friends, it really helped. So… I think that’s one of the basic big things that we could try and provide is, is to ensure that they [young people in care] have like a laptop or phone. And I know, like, I know there’s concerns with the internet and things; I mean, some people get their phones checked. (Young person)

While this difficulty for parents and carers in managing risk online for their children has been identified outside of care (Livingstone et al., 2017; Pescott, 2022), the additional regulations applied to children in care can make this risk management more complex.

### Entering the digital world within the home

Despite the restrictions and the surveillance, the online space could present an opportunity for young people to access support. However, this depended on how well young people, carers and professionals were able to negotiate access to the online space, translate their skills and communication styles between the two spaces and feel able to be open.

Communication online involved significant adaptations. Young people, carers and professionals highlighted the opportunities and limitations of interacting within digital spaces. Some young people felt more comfortable online:

Because it’s online, and I know it sounds weird, I don’t get nervous, or anything … I’ve always been used to online stuff. (Young person)

However, young people also recognised the difference that having audio and visual communication could have on the interaction, and how different tools could be used to communicate more than just words. The need to ‘see other people’ to feel that they are there was evident in this account from one young participant:

The good thing about that, in a way, was that we had cameras on and audio, and we were actually talking, instead of just typing… I think that was quite effective… I mean, just to see other people and see what they were doing, and know that they were there, and not, you know, because you forget, there’s all these other people that exist as well. (Young person)

Prior experiences of young people in care could impact on communications with professionals, complicating forms of online communication. Professionals were also aware that young people may not feel able to be open or visible, but they found it difficult to read the nuances of online interactions:

Because you can’t, by not having them in the same room as you, you can’t pick up on the subtleties, what’s going on… a lot of the young people we work with, they become very adept at covering, um, how they feel. (Practitioner)

While professionals had concerns about their ability to access the paralanguage that is visible in face-to-face settings, they also recognised the needs and the rights that young people have to control how, or if, they choose to engage (see also Featherstone and Bowyer, 2020):

If a young person decides to switch their camera off, which, again, we give them full autonomy to be able to do, I can’t see them. I can’t tell if they’re engaged, I can’t tell if they’re okay… is something we’re talking about affecting them on a personal level? And maybe they need some support. So, it does completely change the dynamic of it. (Practitioner)

Both young people and social care professionals discussed how remote forms of communication engendered autonomy. For example, young people were able to turn off the camera or drop in and out of online conferencing events, depending on their own needs and preferences. This indicates that young people felt they had a choice about whether or not to allow their face and their home space to be shown on screen. As noted in previous sections, young people in care may have limited control over their boundaries in their ‘private’ space. Therefore, how they present in online communication, with the ability to disengage/hang up/switch off video can become a powerful tool for maintaining autonomy.

While this behaviour can be seen as a problem for professionals and carers in terms of the young person’s engagement, it can also be viewed as young people taking control of the terms of their engagement. Some professionals understood the importance of this autonomy as more services transitioned online:

For them, if that’s something they’ve maybe experienced or witnessed that could be something really triggering. And it’s that giving them the safe space to say you can switch your camera off, you can switch your mic off and you can leave the session, but please just let us know if you are so that we can come back and check on you. (Practitioner)

What young people might see as using their autonomy to engage or not may be interpreted differently by caregivers. Carers discussed how remote communications provided opportunities for children and young people to ‘hide’ both physically and metaphorically:

I think for him it’s, it’s a case of he knows he [can] just hide under the sofa, or go up to his bedroom and he doesn’t have to engage. Whereas when somebody’s physically here, there’s more, I suppose, opportunity to have that engagement, whereas on a video call, it’s very easy for them to say, ‘I’m not doing this. I’m going out of the room. I’m going up to my bedroom. I’m going to sit in the bathroom. I’m going to hide under the sofa’, you know? So, I just think it, it’s easier for them to shy away from that. (Carer)

This indicates a tension between the choice of a child or young person as to whether they want to engage and the need that is perceived by carers and professionals for them to engage with the service on offer. This was recognised by young people, and one commented that online schooling could be problematic in terms of safeguarding children at risk when they were not physically seen. In their own experience, online learning had facilitated an opportunity to disengage: ‘I fell quite behind last time, like in the end of Year 12, and being online for, for this time has allowed me to, like sort of hide’ (Young person). Accordingly, online learning enabled a less active engagement with studying as the participant felt less observable and had to rely more on motivating themselves rather than being pushed by their teacher or peer group.

These accounts illustrate the ways in which young people communicate both ‘authentic’ and ‘deceptive’ selves when engaging in remote forms of communication (Lincoln, 2012). In some carers’ accounts, this was presented as an active choice of young people:

When she came off the first one [phone consultation], I said, ‘Well, you lied all the way through that’. And she said, ‘I know’. She said, ‘Well, it doesn’t matter. I don’t know her … I lied to her all the way through’. I said, ‘But how are you going to get help?’… there was a lot of things going on for her … But she just continued to lie to her … So really, when she made the decision to not speak to her, she said to her, ‘Do you mind if I don’t speak to you?’ – we were in the car, by the way – and she said, ‘No, no problem’. And she said … And she actually took control of the conversation and said, ‘To be honest, I’m not really finding this useful’. (Carer)

The child ‘hiding’ can be interpreted as an overt disengagement: an observable form of hiding in plain sight in which the carer and social worker are aware of the child’s intention. However, lying to a social care worker on the phone suggests a misleading presentation of self to others (Goffman, 1959), made easier by remote forms of communication. In the account shared, this seemed to be related to the perceived usefulness (or not) of the attempted intervention and the relationship (or lack thereof) between the young person and the practitioner. In this way, non-engagement could be seen as a prompt for practitioners to change their approach.

Of course, there are always opportunities to tailor what is shared in face-to-face interactions (Goffman, 1959; Ward, 2015; 2016) but the production of an ‘acceptable self’, which requires no support or intervention, may be an easier deception to perform remotely. This was noted in relation to young people attending medical appointments, with the example given about young people with eating disorders:

[The] Dietician’s is one of those things where it needs to be face-to-face … I now don’t get weighed … that’s the whole point on this, it’s a bit silly … people with eating disorders and stuff shouldn’t keep scales in their home … They’re going to want to hide things. That’s why in-person [in person appointments might be better]. It’s a lot easier to hide something over the phone. Like, say with my weight, I could lie and say I’m doing really well, when in fact I’m minutes away from hospitalisation. (Young person)

This disembodied mode of phone interactions enabled a space for the young person to hide the reality of their health condition. As noted by health and social care professionals, this concealment is enabled because of the lack of access to ‘the subtleties’, namely facial expressions and body language. Social work has been described as a ‘visual practice of surveillance and risk identification’ (Dillon, Evans and Wroe, 2021: 291) in which social workers need to ‘see’ the child to assess the level of risk. However, it should be noted that some young people who were actively seeking support reported that medical professionals were able to communicate an element of genuine care and understanding despite the interactions only taking place on the phone. Therefore, the usefulness of remote communication is to some extent dependent on whether young people want, and feel comfortable, to disclose the issues they are facing or wish to keep them ‘hidden’.

## Discussion

The findings of this study have highlighted the complex nature of the home for young people in care. The surveillance and scrutiny that foster and kinship families live with can create an environment in which rules and oversight impact upon the ability of young people to explore digital spaces and access online support. These rules are necessitated by the risk averse nature of child and family social work. However, the accounts of young people, carers and professionals reported here indicate the importance of digital spaces and online communication for care-experienced young people. While the Covid-19 pandemic increased the salience of digital spaces for young people, digital interactions took on more complicated roles as portals to the virtual world within the space of the home, where scrutiny and surveillance are more common than for the general population. As we have demonstrated, this had a wide range of implications for the social lives, therapeutic support and privacy of young people in care.

There is a wider context of instability and trauma for care-experienced young people that indicates that, for some, home does not feel like a safe place. In this study, the lack of private space within the home during the pandemic exacerbated the inability of young people to access confidential support, particularly when the issues raised could be related to their care experience and current living situations. Moving support online may have given young people more autonomy in the ways in which they engaged with services. However, remote interventions did not necessarily consider the settings within which portals to the virtual world were being accessed, who was present and how young people presented themselves. This presentation of self is important as online worlds complicate relationships with confidentiality and offer opportunities for young people to ‘hide’ and oscillate between ‘authentic’ and ‘deceptive’ selves (Lincoln, 2012: 207). This ‘hiding’ can be a demonstration of agency for young people, as in the examples of choosing to turn off a camera, but also a potential risk to their welfare, as in the example of medical appointments, suggesting that ongoing service delivery should have blended components that enable in-person interactions and not simply ones that rely on online contact.

### Reflections for carers and practitioners moving forward

There is existing literature based around the navigation of online spaces for parents (see, for example, Livingstone and Blum-Ross, 2020) and the use of online interventions for young people (Archard et al., 2022). However, this present study highlights the impact of the foster home setting (and the rules that govern it) on care-experienced young people’s autonomy and interaction with digital spaces.

There are challenging questions around how much or how little privacy a young person in care is permitted and to what extent this should be different from young people who are not care experienced. There are valid reasons why social workers and carers may not want a young person to access digital content or engage with specific people, especially related to the perceived vulnerability of children in care online (Fursland, 2010). However, the internet and digital skills are important for personal development (Gustavsson and MacEachron, 2015), and restricting access to digital spaces can limit the development of young people in care.

Guidance and ‘house rules’ that have focused on minimising risk online may not have previously given sufficient weight to the importance of these spaces for young people, prioritising instead the avoidance of any risk in online use. In this study and elsewhere (Engelhardt and Royse, 2022), carers have been clear that they need extra support to understand how to support children and young people to engage safely in the digital world. And yet, less emphasis has been placed on how the physical space of the home impacts the ability of those within it to interact with digital spaces. There is, therefore, a need for guidance to be developed, such as The Fostering Network in Wales’s *Digital Risk Assessment for Children and Young People* and the Internet Matters digital programme (www.internetmatters.org), to support practitioners and carers to enable the development of age-appropriate digital skills. This guidance should help young people in care to navigate the online world safely, confidentially (where necessary) and with a critical eye. This includes a negotiation of *how* young people choose to engage, for example cameras on or off, and how practitioners can support the autonomy of young people while carrying out their safeguarding roles. It is also important to remember that young people might choose not to engage with services for many different reasons. While carers and practitioners might feel it is in the best interest for the young person to engage, their autonomy should be respected and different options sought to support the young person and keep them safe.

### Reflections for policy

Government policy on mental health for care-experienced young people needs to be more sensitive to the needs of young people in their specific environments. The large-scale shift to online delivery risks excluding one of the most vulnerable groups of young people. Key quality standards have not yet progressed to address issues around online intervention (National Institute for Health and Care Excellence [NICE], 2021). As online service delivery is expected to remain a norm, this guidance needs to be developed as a priority, involving the perspectives and recommendations of children and young people, with their rights at the core.

### Implications for research

There were a small number of young people (*n* = 4) included in this study, and all contributed from the perspective of a foster home. While carers spoke from the perspectives of foster and kinship carers, there is a need to explore these issues with young people in different types of care homes, including residential care. While many of the issues raised in this paper may be reflective of the care experience, the settings in which children and young people live are variable, and a broader range should be explored to make robust recommendations.

### Reflections on the methods

The semi-structured qualitative interviews provided an opportunity to explore topics of relevance from the perspectives of multiple stakeholders. This was particularly useful due to the diverse range of experience between practitioners, carers and young people. The added strength of including consultation with stakeholders and a care-experienced interviewer meant that the areas covered were relevant to the overall priorities of stakeholders. The diversity of experience among the participant group meant that the areas covered and the data produced were not all directly related to the research questions. However, this enabled a more nuanced understanding of participants’ subjectivities and generated important data that went beyond the objectives set at the onset of the study.

As noted in the Methods section, the sample was small, specifically in relation to young people. This could be a consequence of the recruitment approach, which restricted recruitment to young people who were engaged with a service. However, given the potentially sensitive nature of the data generated, it was important that only young people who were engaged with a service that could support them were included, so while this is a limitation of the study, it is a necessary one. The second limitation came from the restriction of interviews to online only. This was necessary given the restrictions in place at the time of the study. However, the findings of this study highlight the importance of offering both online and in-person options where possible when conducting research with care-experienced young people.

## Conclusion

This paper has laid a foundation to consider how to balance young people’s rights to privacy and confidentiality, while recognising the pressures on carers to document family life and keep the young people in their care safe from harm. The findings draw on a small sample of qualitative interviews; however, many of the findings align with and contribute to the issues raised in the existing literature base, which remain unresolved. This paper therefore demonstrates a need to undertake further research into conceptualisations of privacy, confidentiality and autonomy within foster and kinship care households. In particular, it would be beneficial to generate more data on household members’ feelings about whether young people in care can be given opportunities for managed risk-taking, such as being able to go online unsupervised. Further exploration of carers’ expectations and fears around young people in care accessing digital spaces could help to inform guidance and support offered to carers. Overall, further research is needed into facilitating effective contact between young people and mental health services through digital spaces, which recognises the specific needs and environment of young people in care and their carers. It is important to move forward and create systems, services and care relationships where young people no longer feel that they ‘wouldn’t want to talk about anything too personal’ in their homes.
